# Treehouse: a user-friendly application to obtain subtrees from large phylogenies

**DOI:** 10.1186/s13104-019-4577-5

**Published:** 2019-08-27

**Authors:** Jacob L. Steenwyk, Antonis Rokas

**Affiliations:** Department of Biological Sciences, Vanderbilt University, Nashville, TN 37235 USA

**Keywords:** Phylogenomics, Phylogenetics, Big data, Tree, Tree pruning, Shiny, Graphical user interface

## Abstract

**Objective:**

Phylogenetic trees that contain hundreds to thousands of taxa are now routinely generated. Retrieving the relationships among a subset of taxa in these large phylogenies can be a challenging or time-consuming task. Addressing this challenge requires the development of tools that facilitate the easy retrieval of subtrees from any user-specified set of taxa in a given phylogeny.

**Results:**

We developed *treehouse*, an open source tool that enables the retrieval of any subtree from a given large phylogeny. With a three-step workflow, *treehouse* successfully allows a user to obtain a subtree from any phylogeny. *Treehouse* can help researchers to explore the relationships among any set of taxa from across the tree of life. *Treehouse* is implemented as a shiny application in the R programming language. *Treehouse* software and usage instructions are publicly available at https://github.com/JLSteenwyk/treehouse.

## Introduction

Evolutionary biology relies on understanding the phylogenetic relationships among sets of genes, traits, and organisms under investigation. However, large phylogenies that contain hundreds of taxa are increasingly becoming inaccessible to researchers interested in the relationships of just a few representatives. For example, some phylogenies are so large that taxon information is often challenging or impossible to visualize and is often excluded [1–4]; similarly, the lengths of many internal branches are often very short and the constraints of displaying a large tree in a letter-sized page make the tracing of relationships among a subset of taxa challenging and unnecessarily time-consuming. These issues will increase in frequency as the numbers of taxa included in phylogenies of genes, metagenomes, genomes, etc. continues to rapidly rise.

To address these issues, we introduce *treehouse*, a user-friendly application with minimal dependencies that facilitates the retrieval of subtrees from any user-specified set of taxa in a given phylogeny. Our simple three-step workflow allows users to obtain subtrees from a curated and growing database of large-scale phylogenetic trees from across the tree of life. Additionally, users may obtain subtrees from their own phylogenies which, can facilitate data exploration and inter-disciplinary collaboration. For easy integration into pre-existing project workflows, subtrees obtained from *treehouse* can be easily be downloaded as a newick file or PDF file that retains branch length information. *Treehouse* enables beginner and expert evolutionary biologists alike to reap the benefits of large-scale phylogenetic projects and use them to test evolutionary-based hypotheses.

## Main text

### Materials and methods

#### Data acquisition

The *treehouse* contains a database of 20 representative large phylogenies from across the tree of life (Table 1).

**Table 1:** Curated phylogenies currently available in *treehouse’s* database

Highest level of taxonomic organization	Taxon or taxa represented	Number of taxa	References
Animals	Birds	198 taxa	[5]
Animals	Birds	48 taxa	[6]
Animals	Insects	144 taxa	[7]
Animals	Mammals	37 taxa	[8]
Animals	Mammals	36 taxa	[9]
Animals	Metazoans	36 taxa	[10]
Animals	Metazoans	70 taxa	[11]
Animals	Vertebrates	58 taxa	[12]
Animals	Worms	100 taxa	[13]
Fungi	*Aspergillus* and *Penicillium*	81 taxa	[14]
Fungi	*Cryptococcus neoformans*	387 strains	[15]
Fungi	Fungi	214 taxa	[16]
Fungi	Agaricomycetes	5284 taxa	[2]
Fungi	*Saccharomyces cerevisiae*	1011 strains	[1]
Fungi	Saccharomycotina	86 taxa	[17]
Fungi	Saccharomycotina	332 taxa	[4]
Plant	Caryophyllales	95 taxa	[18]
Plant	Flowering plants	45 taxa	[19]
Plant	Land plants	103 taxa	[20]
Tree of life	Tree of life	3083 taxa	[3]

#### Description of the software

Using *treehouse* requires the R packages phytools, version 0.6–60 [21], and shiny, version 1.2.0 (https://shiny.rstudio.com/). Dependencies of phytools includes maps, version 3.3.0 (https://cran.r-project.org/web/packages/maps/index.html), and ape, version 5.3 [22]. To present the phylogeny as depicted by the original authors, phylogenies from *treehouse’s* database are rooted. The taxa chosen to root the phylogeny on are inferred from figures presented in the original manuscript or, in the case of phylogenies presented without taxa names, personal communications with the authors. Phylogenies are rooted using phytools’s root() function. Using the list of taxa provided by the user, *treehouse* determines the list of taxa to remove from the phylogeny using the setdiff() function. The resulting list is then used to remove taxa in the phylogeny using phytools’s drop.tip() function. To write out the resulting phylogeny in a newick-formatted text file or display it in a scalable-vector-graphic-formatted pdf file, we use the write.tree() and plot.phylo() functions in Ape, respectively. To create a user-friendly and intuitive user-interface, we used shiny.

### Results

#### A three-step workflow to obtain subtrees

**Fig. 1:**
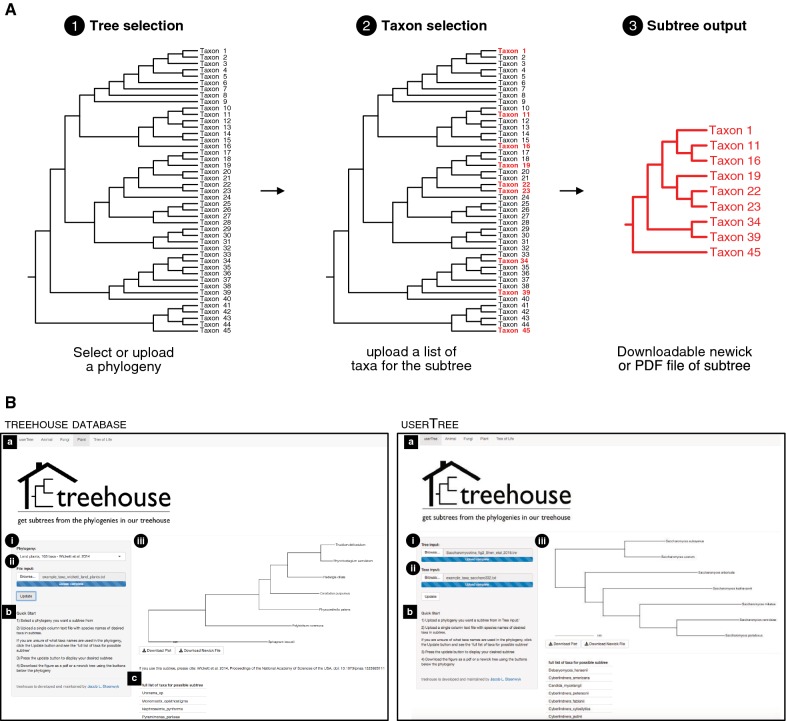
A simple three-step workflow for using *treehouse*. **A** Using *treehouse* requires three simple steps: (1) Tree selection: select a phylogeny from the *treehouse* database or a user-provided phylogeny that you want a subtree for; (2) Taxon selection: upload a list of taxa that a user wants to include in the subtree; and (3) Subtree output: download the newick-formatted text file or scalable-vector-graphic-formatted pdf file of the subtree. **B** *Treehouse’s* user interface features a navigation bar (**a**) to toggle between phylogenies available in *treehouse’s* databases for animals, fungi, plants, and the tree of life (left) and a user provided phylogeny in userTree (right). **b** To enable easy usage of *treehouse,* quick start directions are displayed. **i** A dropdown menu allows for selection of a larger phylogeny to obtain a subtree from when using phylogenies in *treehouse’s* database. When using userTree, a browser function allows a user to upload their own phylogeny. **ii** A browser function allows the user to upload a list of taxa for the desired subtree. **c** A list of all possible taxa in phylogeny is provided

*Treehouse* is designed to have a simple user-interface that guides a user through an intuitive three-step workflow (Fig. 1A) and user interface (Fig. 1B).

1. Tree selection
  A user can choose between five tabs—userTree, Animals, Fungi, Plants, and Tree of Life—located at the top of the user interface (Fig. 1Ba). When using phylogenies from the *treehouse* database, a user selects the desired phylogeny using a dropdown menu (Fig. 1Bi; left). In userTree, a user selects a phylogeny in newick format from their local computer (Fig. 1Bi; right).
2. Selection of Taxa
  A user next uploads a text file containing the single-column list of taxa that they want a subtree for (Fig. 1Bii). Here, each taxon name must be identical to a taxon name in the full phylogeny.
3. Subtree output
  By clicking the ‘Update’ button, the user launches *treehouse* subtree retrieval. The subtree is plotted to the right of the side panel and buttons that allow the user to download a pdf or text file of the subtree are below it (Fig. 1Biii). Lastly, the full set of taxa in the currently uploaded *treehouse* phylogeny is listed (Fig. 1Bc; left).

### Conclusion

*Treehouse* is a simple and powerful tool that facilitates subtree retrieval from large phylogenies.

## Limitations

*Treehouse’s* functionality rests on the performance of one task, namely removing taxa from a phylogeny. To the experienced phylogenetic or phylogenomic researcher, this might seem to be a trivial task but is not so for most users of phylogenetic trees and no other user-friendly methods are available. Thus, we anticipate the ‘typical’ *treehouse* users to be researchers that use phylogenies to form hypotheses but do not routinely infer phylogenies themselves. We also anticipate *treehouse* to be a useful teaching tool.

## Data Availability

All data, materials, and code are publically available at https://github.com/JLSteenwyk/treehouse.
